# Developments in Diagnosis of Visceral Leishmaniasis in the Elimination Era

**DOI:** 10.1155/2015/239469

**Published:** 2015-12-30

**Authors:** Om Prakash Singh, Shyam Sundar

**Affiliations:** Infectious Disease Research Laboratory, Department of Medicine, Institute of Medical Sciences, Banaras Hindu University, Varanasi 221005, India

## Abstract

Visceral leishmaniasis (VL) is the most devastating parasitic infection worldwide causing high morbidity and mortality. Clinical presentation of VL ranges from asymptomatic or subclinical infection to severe and complicated symptomatic disease. A major challenge in the clinical management of VL is the weakness of health systems in disease endemic regions. People affected by VL mostly present to primary health care centers (PHCs), often late in their therapeutic itinerary. PHC physicians face a major challenge: they do not deal with a single disease issue but with patients presenting with complaints pointing to several diagnostic possibilities. Risk exists when some patients having less clinical manifestations are misdiagnosed. Therefore, field based accurate, sensitive, and cost effective rapid diagnostic tools that can detect disease in its mildest form are essential for effective control and reaching the goal of VL elimination. In this review, we discuss the current status and challenges of various diagnostic tools for the diagnosis of VL and assess their application in resource poor settings.

## 1. Introduction

Visceral leishmaniasis (VL) or kala-azar is one of the most neglected poverty related disease with an estimated worldwide incidence of 0.2–0.4 million new cases per year [1]. About 90% of these cases occur in just six countries, including India, Bangladesh, Sudan, South Sudan, Ethiopia, and Brazil [1]. In the Indian subcontinent and East Africa, VL is caused by* L. donovani*, which is transmitted by the sand fly* P. argentipes*, without any known animal reservoir [2], whereas, in Europe, North Africa, and Latin America, it is caused by* L. chagasi* (syn.* L. infantum*) which have both canines and human as reservoirs [3]. More than 100,000 cases occur in India alone every year and the state of Bihar accounts for majority of these cases [4]. However, these figures are official report, mainly based on passive case reporting, and are considered to be an underestimation of the real number of VL cases [5].

VL is clearly a poverty related disease, affecting indeed the poorest of the poor but also blocking the economic development of affected areas. In the year 2000, Thakur described the socioeconomic conditions of a cohort of 938 VL patients from Bihar in India. 75% of them were classified as poor (daily income < US $1) and 82% were engaged in agriculture and/or animal husbandry [6]. VL has recently earned most public attention as one of the neglected diseases globally. In the Indian subcontinent (ISC), three countries affected by VL, India, Nepal, and Bangladesh, aspire to eliminate VL from the subcontinent with a target of bringing down the incidence of VL to < 1 per 10000 population by 2015 through various control measures [7, 8]. One of the important components in this endeavor is decreasing transmission through early diagnosis followed by complete treatment. However, when the control programs succeed in lowering the incidence of the targeted condition, the positive predictive values of the diagnostic tests decrease with more risk of false positives. It is therefore important to ensure that the platform and format of diagnostic technologies are appropriate for the prevalence of infection in the local context. The most important challenge with the control approaches is its long-term sustainability. Sooner or later, primary care settings will need to be reinvolved because passive case finding can only be provided at a large scale and in the long-term by the first-line care givers.

In endemic areas of VL, however, clinical decisions taken by physicians are serial and dichotomized, meaning that for a given syndrome (e.g., fever) the possible diagnoses are explored step by step and with a “yes/no” approach. Hence, when clear signs and symptoms are present, a specific disease is considered and when these are absent the disease is discarded and alternative diagnoses are sought. VL is characterized by a number of complexities, and its clinical features are often confused with other febrile illnesses. Risk of misdiagnosis may exist with patients having less clinical manifestations causing delay in treatment and thus leading to the death of patients. To address this real challenge in clinical care settings, rapid and accurate confirmatory diagnostic test is needed as antileishmanial drugs can cause significant adverse reactions. Accurate diagnostic tools will have a major impact on the ability of countries to estimate accurate disease burden. It would also allow them to track disease trends over time and to determine the effectiveness of future control interventions such as improved diagnosis-treatment algorithms and new vector management strategies. In the following sections, we briefly discuss the currently available diagnostic approaches for VL along with their effectiveness and limitations at the primary health care facilities in disease endemic areas.

## 2. Current Diagnosis of VL and Challenges

VL is characterized by a persistent febrile syndrome, usually associated with splenomegaly that progressively leads to wasting, anaemia, and death due to bleeding or superimposed bacterial infection. Early detection and proper management are crucial for control of this disease. Noninvasive rapid test to be used to diagnose VL and/or as a marker of cure at peripheral health centers could have a great impact on the way VL is managed in endemic communities. A comparative overview of sensitivities and specificities of various diagnostic assays currently in use for VL is presented in Table 1.

### 2.1. Parasitological Diagnosis: Most Specific (Gold Standard) but Less Sensitive

Detection of a parasite is a very specific method and is the first-line approach in VL diagnosis. Microscopic examination of peripheral blood smear or buffy coat is noninvasive first-line test. In the case of negative result, the same procedure is being done on splenic biopsies or bone marrow and remains the most specific method of diagnosis still in practice in the Indian subcontinent and East Africa [9]. Parasitological test with bone marrow aspirate is most frequent in Brazil [10]. However, these procedures are uncomfortable, are potentially dangerous, require considerable skill, and thus are not practical at PHC level.* In vitro* culture of tissue aspirates or blood cells have shown up to 100% sensitivity [11, 12], but these methods are expensive, time consuming, very tedious, and restricted to only dedicated research laboratories. Importantly, parasitological test is the only confirmatory test which exists for relapse patients.

To increase the sensitivity of parasitological diagnosis, antibodies conjugated with fluorescent against surface antigens of the parasite have been carried out in endemic settings in Brazil, Spain, Tunisia, Italy, and Iran [3]. Requirement of a fluorescence microscope restricts the use of IFAT test to referral hospitals.

### 2.2. Immunological Diagnosis: Noninvasive and Rapid

**Figure 1:**
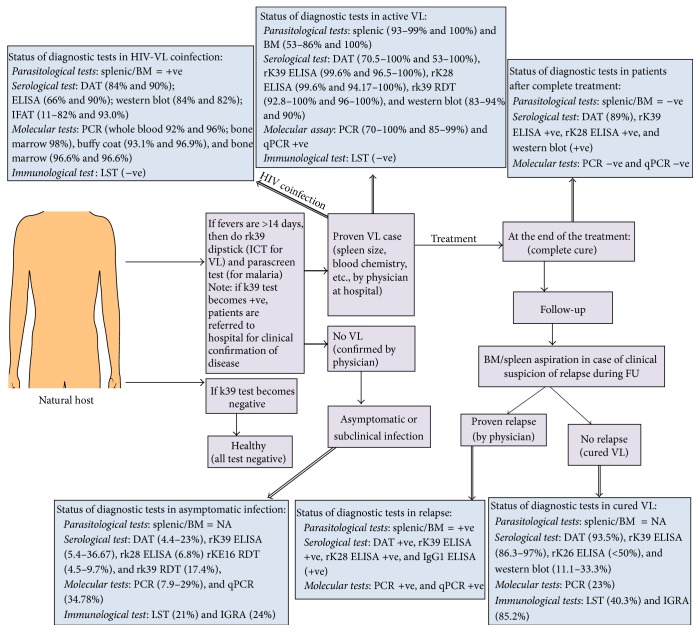
Generalized scheme of disease identification in endemic areas and sensitivity of various diagnostic tests in different pathological condition of VL. +ve: positive; −ve: negative; BM: bone marrow; DAT: direct agglutination test; ELISA: enzyme linked immunosorbent assay; HIV: human immunodeficiency virus; IGRA: interferon-*γ* release assay; LST: leishmanin skin test; NA: not applicable; qPCR: quantitative real time PCR; PCR: polymerase chain reaction; VL: visceral leishmaniasis. Note: in the case of VL and HIV-VL coinfection, both sensitivity and specificity of the diagnostic tests are presented.

Immunological diagnoses are based on the detection of either leishmania antigens or antileishmanial antibodies in the blood samples. Several serological tests have been developed for VL to replace parasitological methods and have been evaluated in different endemic regions with variable results (Table 1). Sensitivity and specificity of such tests depend on the antigens (Figure 1), and, among these, rk39 ELISA and direct agglutination test (DAT) have been extensively validated in endemic areas to document* L. donovani* infection and recommended for VL control programs [13]. However, in Kenya, VL policy specifies that all serologically proven leishmaniasis is confirmed by spleen aspirate, a procedure that can only be performed in referral hospitals [14].

#### 2.2.1. Direct Agglutination Test (DAT)

DAT is a semiqualitative test and highly adopted in field settings that uses microplate with V-shaped well in which coomassie stained whole promastigotes antigen is mixed and serially diluted with patient's serum or blood. If specific antibodies are present, agglutination can be seen after 18 hrs (overnight incubation) with naked eye. DAT has been validated in several countries including India, Nepal, Bangladesh, Sudan, Ethiopia, Kenya, and Brazil [44]. Sensitivity and specificity of DAT vary from 70.5 to 100% and 53 to 100%, respectively [3, 44]. In a recent longitudinal study from India and Nepal, strong associations were found between progression to clinical VL and seropositivity or seroconversion with high DAT titer [13]. Overall, performance of DAT as a diagnostic test is satisfactory, economic (US $1.5–2.5 per test), and independent of the geographical regions [45]. Requirements of electricity, storage of antigens at 2–8°C, multiple pipetting, and need of skilled personnel make it impossible to conduct such diagnostic tests at PHCs.

#### 2.2.2. ELISA

Detection of antileishmanial antibodies through ELISA is very common, and its sensitivity/specificity mainly depends on the antigen used. Previously, ELISA with crude or soluble antigens of promastigotes or amastigotes was used, but cross reactivity was common resulting in giving it least priority in diagnosis. With the advent in technology, several recombinant antigens have been made in VL diagnosis with rK39 being on the top of all recombinant antigens (sensitivity: 67–100%; specificity: 93–100%). However, due to its low sensitivity in Africa [22], new generation fusion antigen k28 was developed with improved sensitivity (92–100% in Sudan) [22] without any changes of its sensitivity in the Indian subcontinent [46]. rK28 ELISA is also useful in diagnosis of cutaneous VL in Brazil [47]. Most importantly, common drawback for all antibody-based detection systems including ELISA is that antileishmanial antibodies persist for 16 years after complete treatment and thus cannot be used as test of cure or relapse [48]. Another limitation of ELISA is that it can be only done in research labs or well equipped hospitals and thus cannot be practical at field setting in endemic areas.

#### 2.2.3. Rapid Diagnostic Test (RDT)

RDT based on the recombinant K39 protein antigen is available and is reproducible, is economic (~US $1.0 per test), is easy to perform, and can give the results within 10 minutes [9, 49–51]. Sensitivity and specificity of rK39 RDT are high in most of the endemic regions (Figure 1). Use of the rK39 RDT is now recommended in combination with a clinical case definition, as a positive rK39 RDT in a healthy subject is not diagnostic of acute disease [52]. In India, about 15–32% of healthy individuals living in the endemic region test positive with the rapid rK39 strip test [52], which is a major drawback of this test as non-VL patients with mimicking illnesses like malaria, enteric fever, and so forth might receive antileishmanial treatment. In India, VL elimination initiative has adopted the rK39 RDT as the main tool, but its limitations are sorely felt [52, 53]. Response of rK39 RDT is less effective in East Africa (Sudan and Ethiopia) demonstrating sensitivities between 70% and 94% [22]. The rK39 strip test performs moderately in South American region (Brazil and Venezuela) where sensitivities and specificities vary from 86 to 100% and 82 to 100%, respectively. Recently, WHO conducted the evaluation of five different immune-chromatographic tests (ICT) utilizing either rK39 or rKE16 on the Indian subcontinent, East-Africa, and Brazil. Result of this study showed the sensitivity between 36.8 and 100% and specificity between 90.8 and 100% [24]. No test was a clear winner across all regions and conditions, but high diagnostic accuracy was shown by rk39 RDT in India and Nepal. Later on, in two subsequent separate studies in India comparing the performance on serum and blood, k39 RDT showed excellent agreement with high diagnostic accuracy [25, 26]. Importantly, in areas where K39 dipsticks are not readily available and parasitological diagnosis (e.g., spleen aspiration) is not feasible, clinicians still tend to rely on clinical diagnosis with consequent overdiagnosis and misallocation of already stretched resources. These RDT tests however cannot discriminate between current, subclinical, or past infections and are useless for diagnosis of relapses and as prognostic (cure) tests.

More recently, rK39 RDT was tested and evaluated on urine samples in India and Bangladesh with sensitivity of 96.1–100% and 95%, respectively [27–29, 54]. Excellent diagnostic performance of rK39 RDT was also shown with saliva samples from India and Tunisia [23, 55]. Performance of rK39 strip test in HIV positive and parasitological confirmed VL patients was also tested, which showed 77% sensitivity [56]. More extensive studies are needed to establish the rK39 RDT for diagnosis of VL using saliva or urine samples and make it also practical in the field condition for the diagnosis of HIV-VL coinfection.

#### 2.2.4. Latex Agglutination Test (KAtex)

An antigen detection test would in theory be considered more specific than antibody-based serological tests [57]. Antigen detection diagnostic tools are required as a means of identifying symptomatic infections in immunocompetent and immunocompromised patients (e.g., diagnosis of primary VL in Sudan, where rK39 RDTs lack sensitivity; diagnosis of relapse cases) and as an indicator of cure. Antigenaemia is a predictable feature of VL, and it is also demonstrated by the efficacy of the KAtex test to detect antigens in urine. KAtex is the only commercially available diagnostic test that has been developed for detection of 5–20 kDa glycoprotein in the urine of VL patients [9]. KAtex is an easy, rapid, and field applicable test with high specificity; however, sensitivity was unsatisfactory and variable in the studies conducted in Indian subcontinent and East Africa [32, 58, 59]. KAtex is 85.7% sensitive in HIV-VL coinfected patients and showed potential as prognostic test [60], but low specificity in immune-competent individuals is a major limitation [61]. The fact that KAtex is noninvasive technique and urine can be collected more easily than blood makes it more acceptable in nonsymptomatic individuals and would allow a longer follow-up. However, its low sensitivity and requirement to boil the urine for five minutes to avoid false positive reactions which affects the reproducibility of this test limit the use of KAtex in peripheral health facilities.

#### 2.2.5. Leishmanin Skin Test and Whole Blood Assay

Montenegro test or leishmanin skin test (LST) measures the delayed type hypersensitive reaction. It is very useful test in the case with cutaneous leishmaniasis (CL) where healing lesions are primarily present. In the case with VL, it is mostly used along with serological markers in endemic areas. Though it has very little diagnostic value in VL (typically negative due to anergic state), it is very useful in VL epidemiology [62]. A GMP-grade* L. donovani* antigen is unavailable, and intradermal administration followed by 48 hrs of test reading is unpractical at PHCs. Whole blood IFN-*γ* release assays (IGRA) have recently been introduced as an alternative to the LST, but clinical application of IGRA in VL diagnosis or treatment is not yet fully established [31].

### 2.3. Molecular Diagnosis: Highly Sensitive and Specific

Although considerable scientific progress has been made over the past decade including the genome sequencing of* L. donovani*, these have not had any impact so far on the quality of clinical care for VL in the field. Though diagnostic accuracy of molecular tests is excellent in laboratory based evaluations, their cost and their clinical benefit when applied in resource-constrained settings are still in debate. So far, various molecular detection methods targeting specific DNA and/or RNA genes have been developed [15]. Polymerase chain reaction (PCR) and real time quantitative PCR (qPCR) are most rewarding gene amplification technique being rapid and quite sensitive but are not feasible in the field settings. Furthermore, very few assays have been validated on broad range of clinical samples, and none of them has become a reference tool in VL diagnosis (reviewed in [63]). Nonetheless, it is currently not very clear how such innovative devices can be meaningfully applied within the health system context of VL endemic areas. However, many researchers claim that when VL control program succeeds in lowering the incidence of infection, these molecular tests will be then an instrumental in maintaining the sustainability of control program by detecting the infection at very low levels.

## 3. Diagnosis of Asymptomatic or Subclinical Infection: A New Challenge in Endemic Areas

Chemotherapy alone as VL control tool is limited by the fact that only sick people will be treated. There are asymptomatic carriers ranging between 1 : 2.4 in Sudan [64], 4 : 1 in Kenya [65], 5.6 : 1 in Ethiopia [66], 18 : 1 in Brazil [67], 50 : 1 in Spain [68], 4 : 1 in Bangladesh [69], and 8.9 : 1 in high-endemic villages of India and Nepal [70, 71], constituting probable parasite reservoirs for sand fly vector (reviewed in [63, 72]). A recent mathematical model suggests a major role of these asymptomatic infections in driving transmission of human VL in ISC [73]. Strong evidence that these infections are real comes from studies in which parasites could be cultured from blood of healthy donors [74] or detected by PCR [19, 75]. Diagnostic tests for asymptomatic infections are therefore needed in order to identify possible hotspots of transmission in endemic areas. Invasive methods to demonstrate the presence of parasites are unethical in asymptomatic individuals; there is therefore no gold standard. A number of studies have used DAT or rK39 based serological tests to document infection; other studies have used seroconversion for one or more tests as marker of infection [20]. Currently, status of asymptomatic infection can be defined in several ways: culture positive, PCR and serology positive, or marker of cellular immune response like IGRA test (Figure 1). Moreover, there is little agreement between the different tests when applied cross-sectionally on the same population group [76]. Serologic testing (e.g., DAT, rk39 ELISA, and western blot assays) is generally assumed to detect recent infection, but the length of time serology remains positive, and whether this differs between VL patients and subclinically infected individuals (as seems likely based on the magnitude of the titers) is not known with certainty. In the absence of a gold standard it is hard to know whether these seropositives who remained healthy were truly infected with* L. donovani* or whether the serology results were just false positive results or prior infection that cleared [63]. The robustness of these test measures could also be influenced by other factors including handling variability and storage practices. Though serological tests are frequently used to measure* L*.* donovani* infections, there is little published data regarding their reproducibility when applied to asymptomatic persons.

Xenodiagnosis is the most direct way and only proof of study to ascertain the infectivity of such asymptomatic infections in disease transmission (reviewed in [63]). Molina et al. used xenodiagnosis as a method to diagnose VL infection in HIV infected patients. Even asymptomatic patients in early stages of HIV infection were able to infect sand flies [77]. In a later study they hypothesize that such patients could trigger a new transmission route of VL in Spain, where the natural host of the disease is the domestic dog [78]. If asymptomatically infected persons can also be a source of transmission there is obviously a need to reconsider certain aspects of the VL control strategy. Therefore, more extensive studies are required in this area in order to provide the epidemiological and clinical evidence that could more broadly inform VL control programs and facilitate the development of novel diagnostic aids.

## 4. Diagnosis of PKDL: An Unresolved Mystery

In a common complication of VL, particularly in Sudan, and to a lesser extent Ethiopia, patients may develop a chronic form called “post-kala-azar dermal leishmaniasis,” or PKDL [79], which occurs within weeks to a few months following treatment, in up to 50–60% of people who have recovered from VL. In the ISC, 10% of VL patients go on to develop PKDL after an interval of 6 months to 4 years [80]. Clinical features consist of hypopigmented macules and/or diffuse infiltration, papules, nodules, or plaques and can be confused with other skin disorders. Except from cosmetic disfiguration, these patients do not suffer from any physical handicap. Although mortality from PKDL is low, PKDL patients represent a largely neglected reservoir of infection that perpetuates anthroponotic* L. donovani* disease in India. Though PKDL in Sudan and in India are both due to* L. donovani*, Sudanese PKDL frequently self-heals (84% in 1 year [79]) whereas Indian PKDL takes longer time to self-heal.

The tools for diagnosis of PKDL are inadequate. In endemic areas, clinical sign and symptoms, along with a previous kala-azar episode and positive antibody tests (e.g., rK39 RDT), are being used to diagnose PKDL without any parasitological confirmation (reviewed in [81]). However, this approach may not be accurate enough as ~10% of PKDL patients have no history of VL and positivity of serological tests up to several years after treatment [82]. Skin slit smear microscopy is the only confirmatory test but is very painful and impractical with macular lesions [83]. LST have low sensitivity, and it is hard to culture parasite due to contamination [82]. Nested PCR is highly sensitive; and recent development of kDNA based quantitative PCR has shown to be efficacious in diagnosis of PKDL [84]. However, these molecular tests are very costly and need sophisticated laboratory to perform. There is a huge gap in treatment of PKDL, and treatments with Ambisome have side effects [80]. Therefore, rapid noninvasive point of care diagnostic tests is urgently required as focal VL outbreaks have been linked to an index case of PKDL [85].

## 5. Diagnosis of HIV-VL Coinfection: Time for Concerted Action

The emergence of HIV and its association with VL poses challenges as how best to diagnose and treat patients presenting with HIV-VL coinfection. The actual number of documented cases of HIV-VL coinfection in India is underestimated due to problems in recognition, reporting, and diagnosis. Patients with HIV-VL coinfection represent an important but largely neglected reservoir of parasites, and focal reemergence of VL have been linked to an index case of HIV (reviewed in [86]). Sensitivity and specificity of various diagnostic methods for HIV-VL have been reviewed by Deniau et al. [87] and Cota et al. [88]. Serological assays are considered not accurate, since the majority of these patients often do not exhibit detectable levels of antibodies. Parasitemia is higher in HIV coinfection [89], thus direct detection of parasite or its component in blood by PCR or qPCR is increasingly used not only for diagnosis but also for the follow-up of the patients during and after treatment, but these tests are often not readily available in poor health care settings (reviewed in [88]). At present, there is not any clear evidence to support recommendations on serological or molecular diagnosis of HIV-VL (Figure 1). Consequently, diagnosis often relies on invasive spleen or bone marrow aspiration or with a combination of RDTs used in a diagnostic algorithm.

## 6. Conclusion

The most important step in VL control is to knock out the last case by employing effective strategies. This can be only possible with availability of rapid and cost effective diagnostic test in disease endemic regions in order to enable the physicians to make accurate therapeutic decisions as arsenal of antileishmanial drugs is limited and is frequently associated with adverse events [90]. Diagnostics needs to be considered broadly and concerns a range of applications like infection, disease, severity, or response to treatment. Visualization of amastigote in suspected VL using microscopy is still the classical confirmatory diagnostic test for VL but is not practical in the endemic areas. A number of less invasive serological tests like DAT, rK39 ELISA, and molecular tests, for example, PCR, are becoming more attractive now, but, at present, these tests are confined to the laboratory. rK39 dipstick or ICT, despite the variability initially observed among different producers and countries, seems to be the first choice for decentralized diagnosis of VL with a good sensitivity and specificity. However, due to some limitations, it must be used in combination with a standardized clinical case definition. No tests are currently available that can detect asymptomatic* L. donovani* infection or predict progression of infection to clinical VL disease. Diagnosis of HIV-VL coinfection will be another problem in coming years. Last but not least, there is a clear need to bridge the gap between current practices in the field of clinical management of VL and available technology and research efforts for VL diagnostics.

## Figures and Tables

**Table 1:** Sensitivity and specificities of various tests in VL diagnosis.

	Methods	Sample used	Test time	Skill level required	Sensitivity (%)	Specificity (%)	References
Parasitological diagnosis	Splenic aspiration	Spleen tissue	Hours	High	93–99	100	[15]
	Bone marrow aspiration/biopsy	Bone marrow	Hours	High	53–86	100	[16, 17]
	Lymph node aspiration	Lymph	Hours	High	53–65	100	[3, 16]
	Culture	Spleen or bone marrow	Days	Medium	97–100	100	[11].

Immunological diagnosis	IFAT	Serum/plasma	Hours	High	80–100	96–100	[18]
	Direct agglutination test	Serum/plasma	Days	Medium	94.80	97.10	[13, 19–21]
	ELISA	Serum/plasma	Hours	Medium	93–100	97–98	[21, 22]
		Saliva	Hours	Medium	83.30	88.6–100	[23]
	Immunochromatic strip test	Serum	Minutes	Low	96.3–100	90.1–100	[24–26]
		Blood	Minutes	Low	96–100	90.8–100	[25, 26]
		Saliva	Minutes	Low	82.50	84.6–91.48	[23]
		Urine	Minutes	Low	96.40	66.2–100	[27–29]
	Immunoblotting assay	Serum/plasma	Hours	Medium	83–94%	90%	[30]
	IFN-*γ* release assay (IGRA)	Whole blood	Days	Medium	80–85	100	[31]
	KAtex test (KAtex)	Urine	Hours	Medium	48–87	89.00–100	[32, 33]

Molecular diagnosis	PCR	Whole blood	Hours	High	70–100	85–99	[34]
		Buccal swab	Hours	High	83.00	90.56	[35]
		Urine	Hours	High	88.0	100	[36]
		Bone marrow	Hours	High	95.30	92.60	[37]
	PCR ELISA	Whole blood	Hours	High	83.90	100	[38]
	qPCR	Whole blood	Hours	High	91.3–100%	95.0–100%	[39]
	Oligo C-test	Whole blood	Hours	High	96.2	90.0	[40, 41]
		Lymph node	Hours	High	96.8	NA	[40]
		Bone marrow	Hours	High	96.9	NA	[40]
	LAMP	Whole blood	Hours	Medium	83.0	98.0	[42]
		Buffy coat	Hours	Medium	90.7	100	[43]
